# Highly divergent 16S rRNA sequences in ribosomal operons of *Scytonema hyalinum* (Cyanobacteria)

**DOI:** 10.1371/journal.pone.0186393

**Published:** 2017-10-26

**Authors:** Jeffrey R. Johansen, Jan Mareš, Nicole Pietrasiak, Markéta Bohunická, Jan Zima, Lenka Štenclová, Tomáš Hauer

**Affiliations:** 1 Department of Biology, John Carroll University, University Heights, Ohio, United States of America; 2 Department of Botany, Faculty of Science, University of South Bohemia, České Budějovice, Czech Republic; 3 Institute of Hydrobiology, Biology Centre of the CAS, v.v.i., České Budějovice, Czech Republic; 4 Centre for Phycology, Institute of Botany of the CAS, v.v.i., Třeboň, Czech Republic; 5 Plant and Environmental Sciences Department, New Mexico State University, Las Cruces, New Mexico, United States of America; INRA, FRANCE

## Abstract

A highly divergent 16S rRNA gene was found in one of the five ribosomal operons present in a species complex currently circumscribed as *Scytonema hyalinum* (Nostocales, Cyanobacteria) using clone libraries. If 16S rRNA sequence macroheterogeneity among ribosomal operons due to insertions, deletions or truncation is excluded, the sequence heterogeneity observed in *S*. *hyalinum* was the highest observed in any prokaryotic species thus far (7.3–9.0%). The secondary structure of the 16S rRNA molecules encoded by the two divergent operons was nearly identical, indicating possible functionality. The 23S rRNA gene was examined for a few strains in this complex, and it was also found to be highly divergent from the gene in Type 2 operons (8.7%), and likewise had nearly identical secondary structure between the Type 1 and Type 2 operons. Furthermore, the 16S-23S ITS showed marked differences consistent between operons among numerous strains. Both operons have promoter sequences that satisfy consensus requirements for functional prokaryotic transcription initiation. Horizontal gene transfer from another unknown heterocytous cyanobacterium is considered the most likely explanation for the origin of this molecule, but does not explain the ultimate origin of this sequence, which is very divergent from all 16S rRNA sequences found thus far in cyanobacteria. The divergent sequence is highly conserved among numerous strains of *S*. *hyalinum*, suggesting adaptive advantage and selective constraint of the divergent sequence.

## Introduction

Small subunit rRNA gene sequence data have become critical for understanding microbial evolution, definition of taxa, estimating metagenomic diversity in localized environments, and estimating total microbial diversity on the planet [1–3]. It is considered by many to be the best gene locus for studying evolutionary history because it is universal in prokaryotes and eukaryotes, is stable, has informative variable regions, and has extensive representation in sequence databases, as well as a purportedly low incidence of horizontal gene transfer (HGT). In bacteria, the more highly variable flanking region, the 16S-23S internal transcribed spacer (ITS) region can be amplified in the same PCR reaction and provides additional resolution of microdiversity and species limits [4–6]. In some cyanobacteria, primary sequence and secondary structure of ITS regions have been used to delineate and name both phenotypically distinct species as well as cryptic species [7–10].

Recently, the utility of the 16S rRNA gene in creating phylogenies and in estimating biodiversity has been questioned due to two related discoveries. First, over 80% of the prokaryote genomes sequenced have more than one operon, with copy numbers from 2 to 15 in bacteria and 2 to 4 in archaea [11,12]. In most cases, the sequence of these different ribosomal operons is highly similar in the 16S rDNA, ostensibly due to gene conversion [13,14]. However, in the last 25 years instances in which operons are highly divergent within a single genome have been discovered [15–18]. Apart from instances of gene truncation (which produces pseudogenes), the most divergent operons are those that have large insertions or intervening sequences [11]. Divergence in which secondary structure of the 16S rRNA molecule is retained, but exceeds the recommended species boundary of 1.0–1.3% divergence was recently catalogued in 14 of 568 species surveyed. Of these divergent operons, only seven had divergences >2.0%; *Thermoanaerobacter* with 11.6% divergence due to two large insertions, and *Halosimplex carlsbadense*, *Haloarcula marismortui*, *Natrinema* sp., *Hamaeophilus influenza*, *Veillonella* sp., and *Clostridium cellulolyticum*, with 6.7%, 5.63%, 5.0%, 2.75%, 2.5% and 2.07% divergence, respectively, due to localized diversity [11,18,19]. Second, HGT is thought to confuse the phylogenetic signal in sequence data. Some researchers downplay the problem of HGT by noting it is most common in genes not used to reconstruct phylogeny [1] and single gene 16S rRNA phylogenies are still very robust [3]. Others express concern due to the discovery of HGT in the ribosomal genes [20–23]. In particular, Yap et al. [24] give compelling evidence that an entire ribosomal operon was transferred laterally to *Thermomonospora chromogenia* from another species, likely *Thermospora bispora* or a closely related taxon.

We here report the first instance of macroheterogeneity in the 16S rRNA gene and associated 16S-23S ITS region, 23S rRNA gene, and 5S rRNA gene of ribosomal operons in a member of the phylum cyanobacteria, *Scytonema hyalinum*. We present evidence to show that 1) localized diversity possibly indicative of HGT of an entire ribosomal operon exists in this cyanobacterial species cluster, 2) this putative HGT is an event that occurred prior to speciation within this species cluster, 3) secondary structure of the ribosomal rRNA of both subunits remains intact in the horizontally transferred operon, 4) gene conversion has not reversed the heterogeneity introduced by HGT, indicating that the introduced gene may increase fitness at the genus level.

## Materials and methods

### DNA isolation and sequencing

Cyanobacterial strains were cultivated on Z8 [25] agar-solidified medium (1.5%) at 22°C in a 12:12 light:dark cycle. Total genomic DNA of strains obtained from China (CXA), South America (ATA), the Hawaiian Islands (HA), and selected strains from North America (CMT, WJT, CNP, HAF, HTT, FI) were extracted using the UltraClean Microbial DNA Isolation Kit following the manufacturer’s protocol (Mo Bio Laboratories, Carlsbad, California, USA). The biomass of the remaining strains was dried for 48 hours over silica gel and pulverized in a Mixer Mill MM200 (Retsch, Haan, Germany) laboratory mill with wolfram carbide beads (3 min, 30·s^-1^). Total genomic DNA was isolated following a modified xanthogenate-sodium dodecyl sulfate buffer extraction protocol with addition of 3% polyvinyl polypyrrolidone and polyethylene glycol-MgCl_2_ precipitation [26]. The PCR amplification of three conserved protein-coding genomic loci was performed using published protocols without modification (fragment of the DNA-directed RNA polymerase gamma subunit gene *rpo*C1 [27]; fragment of the RuBisCO operon *rbc*LX [28]; fragment of the nitrogenase molybdenum-iron protein alpha chain *nif*D [29]); PCR products were sequenced directly using the same primers. Amplification of a ca. 1600 nucleotide PCR product representing the 16S rRNA and 16S 23S ITS ribosomal rRNA gene region followed procedures outlined in [4, 30]. PCR products were purified and cloned using the Stratagene (Agilent Technologies, La Jolla, California, USA) or pGEM®-T Easy (Promega Corp., Madison, WI, USA) vector systems. The plasmids containing inserts were purified from 8–20 *E*. *coli* colonies and sequenced until multiple rRNA operons were obtained.

To recover Type 1 and Type 2 operons missed by cloning due to biased PCR amplification of the two divergent paralogues, and to be able to sequence the initial part of the 16S r RNA gene, we designed reverse primers specific for each of the two operon types: HY1R (5’-GGA ATA ACG ACT TCG GGC AAA ACC AA-3’) for Type1 and HY2R (5’-AGG GTA ACG ACT TCG GGC GTG ACC AG-3’) for Type 2. The sequences of these primers were 100% conserved in operons previously recovered using the cloning strategy. The PCR using 16S27F primer [31] and HY1R/HY2R primers, amplifying the first ~800 bp of the 16S rRNA gene, included an initial denaturation step at 94°C for 5 min, followed by 35 cycles of 40 s at 94°C, 45 s at 55°C, and 1 min 20 s at 72°C, and a final elongation step for 7 min at 72°C. PCR products were directly sequenced using the same primers.

To recover nearly full sequences of the rRNA operons of both types in the strain *S*. *hyalinum* HTT-U-KK4 we matched the 16S+ITS rRNA sequences collected earlier with sequences obtained using several PCR reactions with overlapping products: (i) the leader region of the rRNA operon and the partial 16S rRNA gene using primers 16S promoter [32] and the specific HY1R/2R primers; (ii) the nearly complete 23S rRNA gene using primers KP36F/VC2763R and protocols according to Haugen et al. [33]; (iii) the central part of the operons using reverse complement primers to HY1R/2R and the KP591R reverse primer [33]; (iv) the terminal part of 23S rRNA and partial 5S rRNA using a primer combination WL2419F [33] and 5SR [32]. All PCR reactions were performed with an initial denaturation step at 95°C for 5 min, followed by 36–40 cycles of 45 s at 95°C, 45 s annealing at 52°C, 30 s elongation per each 500 bp at 72°C, and a final elongation step for 10 min at 72°C. PCR products were cloned as previously and sequenced using the T7promoter and SP6R primers included in the vector. The 23S rRNA was additionally sequenced using internal primers KP798F, WL1608F and WL2242F [33].

Two strains of *S*. *hyalinum*, HA4185-MV1 and WJT9-NPBG6B, were selected for draft genome sequencing. Total genomic DNA was amplified from single filaments using multiple displacement amplification, and sequenced using a Pair-End genomic library with ~350 bp average insert length and 250 bp sequencing reads on the Illumina Mi-Seq platform (Illumina, Inc., San Diego, CA, USA). The protocols exactly followed the procedures described in detail previously [34]. The data were assembled using default settings in CLC Bio Genomics Workbench v. 10 (Qiagen Bioinformatics, Redwood, CA, USA) and inspected for rRNA operons only.

All sequences were deposited in the NCBI database under accession numbers KY365438-512, KY407662-663, KY416993-KY417088, KY423285-332, and MF574178-181. A nearly complete genome scaffold of *Scytonema hyalinum* HK-05 was sequenced by other authors (NCBI accession AP018194), and reported with seven separate plasmids, including a 16.3 kb plasmid containing a full ribosomal operon that is discussed in this paper (NCBI accession AP018198).

### Sequence analysis

For phylogenetic analysis, the 16S rRNA sequences from individual operons obtained using the cloning strategy and the PCR with specific HY1R/HY2R primers that were 100% consistent in the overlapping regions were merged to cover nearly the entire 16S rRNA gene (missing only the first 27 nucleotides). DNA sequences were aligned together with representative sequences of major clades of heterocytous cyanobacteria with sequenced whole genomes, and a set of close BLAST hits (16S rRNA gene). Sequences of the three protein-coding loci (*rpo*C1, *rbc*LX, *nif*D) were aligned using MAFFT v. 7 [35] and manually checked; from the RuBisCO operon only the coding regions were included in the phylogenetic analysis. A Maximum Likelihood (ML) phylogenetic analysis in RaxML v.8 [36] employing GTR+I+G substitution model was run with each of the protein-coding loci separately, with 1000 bootstrap pseudo-replications. Resulting phylogenies were manually checked to reveal and eliminate taxa exhibiting incongruent positions in individual gene trees. The three resulting concordant matrices were then concatenated prior to the final analysis. The 16S rRNA gene sequences were aligned using ClustalW, and manually corrected to preserve conserved secondary structure. Phylogenies inferred from the rRNA and concatenated protein-coding genes were reconstructed using Bayesian Inference (BI), ML, and Neighbor-Joining (NJ) methods. The BI calculation in MrBayes 3.2.6 [37] involved two runs of eight Markov Chains Monte Carlo (MCMC) for ≥1,000,000 generations, sampled each 100 generations until the convergence criterion reached a value <0.01. The first 25% of the sampled data was discarded as burn-in. ML analysis was performed as previously. The best-fitting nucleotide substitution models for ML-based methods were estimated using Akaike Information Criterion (AIC) values (jModelTest 2.1.6.; [38,39]) for each of the loci separately. For the 16S rRNA data set, a GTR+I+G model was selected and applied in both ML and BI analyses. For the protein-coding alignments, submodels from the GTR family were selected by the software for each of them (*nif*D: TrN+I+G; *rpo*C1: GTR+I+G; *rbc*LX: Tim2+I+G). RaxML and MrBayes currently provide only few options for calculation with GTR models, and the actual best-fitting model for the data would presumably be even more complicated (including separate models for each codon position, etc.). Thus, in RaxML we used the most general GTR+I+G model (separate for each partition/locus), and in MrBayes we compared two extremes–a partitioned GTR+I+G model versus default settings (F81 non-partitioned model). Default settings in MrBayes resulted in a topology more congruent with the 16S rRNA tree, which was then used in the main text. ML and BI analyses were run using the CIPRES supercomputing facility [40]. The NJ analysis was run in SeaView v. 4 [41] using the BioNJ algorithm [42] and (default) Jukes-Cantor substitution model, with 1000 bootstrap pseudo-replications. The alignments and phylogenetic trees were deposited in Dryad, at DOI:10.5061/dryad.6s386.

Sequence identities were calculated as 100*(1– (p-distance)), with p-distance obtained using the SHOWDIST command in PAUP version 4.0b10. Percent sequence divergence is simply 100*(p-distance). Determination of genospecies within the *Scytonema* strains was estimated using the following criteria 1) If two strains had 16S rRNA identity ≤ 98.7% in either operon, they were considered separate species [3]; 2) If strains were phylogenetically separated, they were considered to be separate species; and 3) named morphologically different species (e.g. *Scytonema arcangeli* and *Scytonema hyalinum*) were accepted as separate species based on phenotypic traits even if molecular support for their separation was weak. This is a conservative estimate of species, and detailed analysis of both morphology and 16S-23S ITS p-distance and secondary structures could reveal additional unnamed cryptic species [7,43]. We consider this detailed taxonomic analysis and revision beyond the scope of this paper.

The secondary structure estimations were made following the models for the 16S rRNA, 23S rRNA, and 5S rRNA molecules published for *E*. *coli* on the Comparative RNA site (CRW) [44]. Modifications for longer or shorter helices were required for selected helices, and the terminal structures of the helices were determined in Mfold 3.2 [45]. In instances where helices with 1–2 mismatches in *E*. *coli* could pair in *Scytonema* with canonical base pairings, the helices were closed. The secondary structure figures were assembled manually in Adobe Illustrator CS5 version 15.0.0.

## Results and discussion

**Fig 1:**
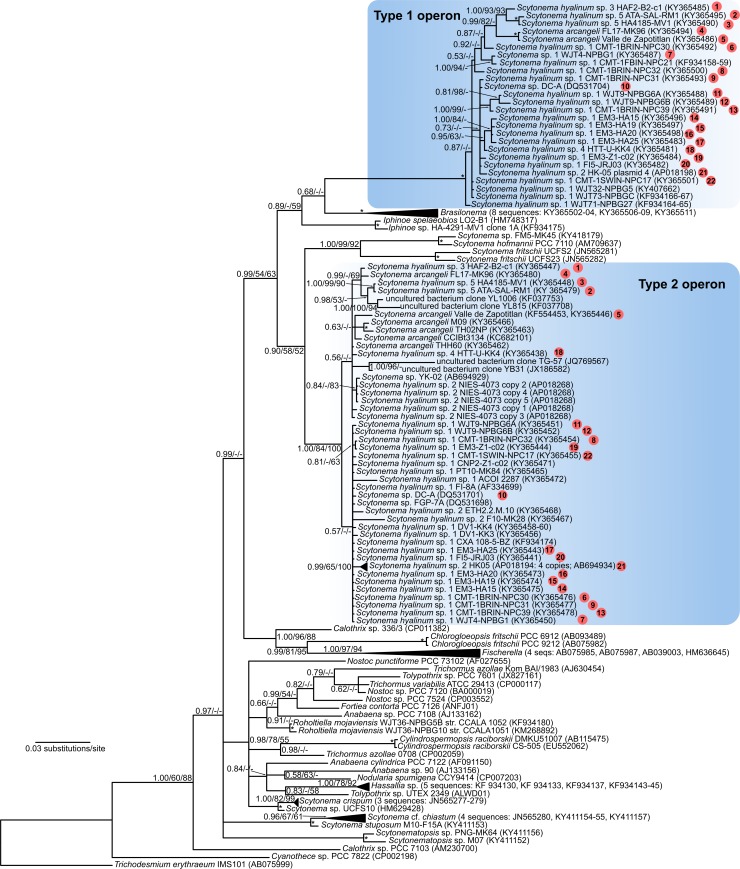
Phylogenetic tree based on 16S rRNA data. Sequences of the *Scytonema hyalinum* species cluster were generated using a combination of cloning strategy and PCR with specific primers designed for each of the two divergent operon types (for details see Materials and Methods). The clades corresponding to rRNA operon Type 1 and Type 2 are shown in shaded boxes. The operon Type 1 sequences form a long branch in the tree due to high dissimilarity to all available 16S rRNA sequences of cyanobacteria. Circles with numbers inside indicate strains in which both operon types were recovered. The tree is based on Bayesian Inference; branch supports ≥50% are given at the nodes in this shape: Bayesian Inference/Maximum Likelihood/Neighbor-Joining. Asterisks indicate nodes with ≥95% support from all methods.

*Scytonema hyalinum* possesses five ribosomal operons as evidenced by the heterogeneity observed in multiple cloned PCR amplicons (S1 Table) and two recently sequenced genomes of *Scytonema* sp. (NIES 4073 and HK-05), each of which have five operons. Four operons are very similar to each other in the 16S rDNA (>99.5%) and distinguishable only by the divergent 16S-23S ITS flanking the 3’ end of the 16S rRNA gene (S1 Table). These operons, which we designate as Type 2 operons (Fig 1) are highly similar to other heterocytous taxa outside of the *S*. *hyalinum* cluster (up to 96% sequence identity (SI) to 16S rDNA in *Scytonema sensu stricto* and *Brasilonema*, 94–95% SI to heterocytous taxa outside of the Scytonemataceae). The fifth operon, designated as the Type 1 operon (Fig 1) is divergent from the others, being only 91.0–92.7% similar to the Type 2 operon when comparison is made within strains. Among divergent operons in prokaryotes observed thus far, this is the highest divergence seen among heterogeneous 16S rRNA genes that do not have insertions or truncation (7.3–9.0%). These divergent operons were obtained by several workers in several labs, in strains from four continents and Pacific islands, isolated over a period of over 15 years. Yarza et al. [3] state that an SI <94.5% is strong evidence of different genera, while SI <98.7% is strong evidence of different species. If we had not cloned the PCR amplicons from strains or obtained sequences from environmental DNA, so that only a single sequence for each strain/lineage was obtained, we would have concluded that we had two phylogenetically well-separated genera (Fig 1), with at least 6 species in the Type 2 genus, and at least 2 species in the Type 1 genus. With an understanding of macroheterogeneity in the operons, we conclude that six distinct genospecies are present within a single genus (S1 and S2 Tables).

The ITS regions of all Type 1 operons differ in a number of ways from the ITS regions of all of the Type 2 operons of *S*. *hyalinum* as well as all available heterocytous cyanobacteria. The marked differences include: i) the first three bases of the ITS are AAC, when in almost all cyanobacteria these bases are TTT, TTA, or TAT; ii) the spacer between D2 and D3 regions is 10–11 nucleotides, compared to 4–6 nucleotides in Type 2 operons; iii) the D3 region is GGTAY, which differs from GGTTC in all Type 2 operons; iv) the D4 is longer and differs in sequence; v) the D5 is shorter, 6 nucleotides compared to 15–16 (S1 Table).

**Fig 2:**
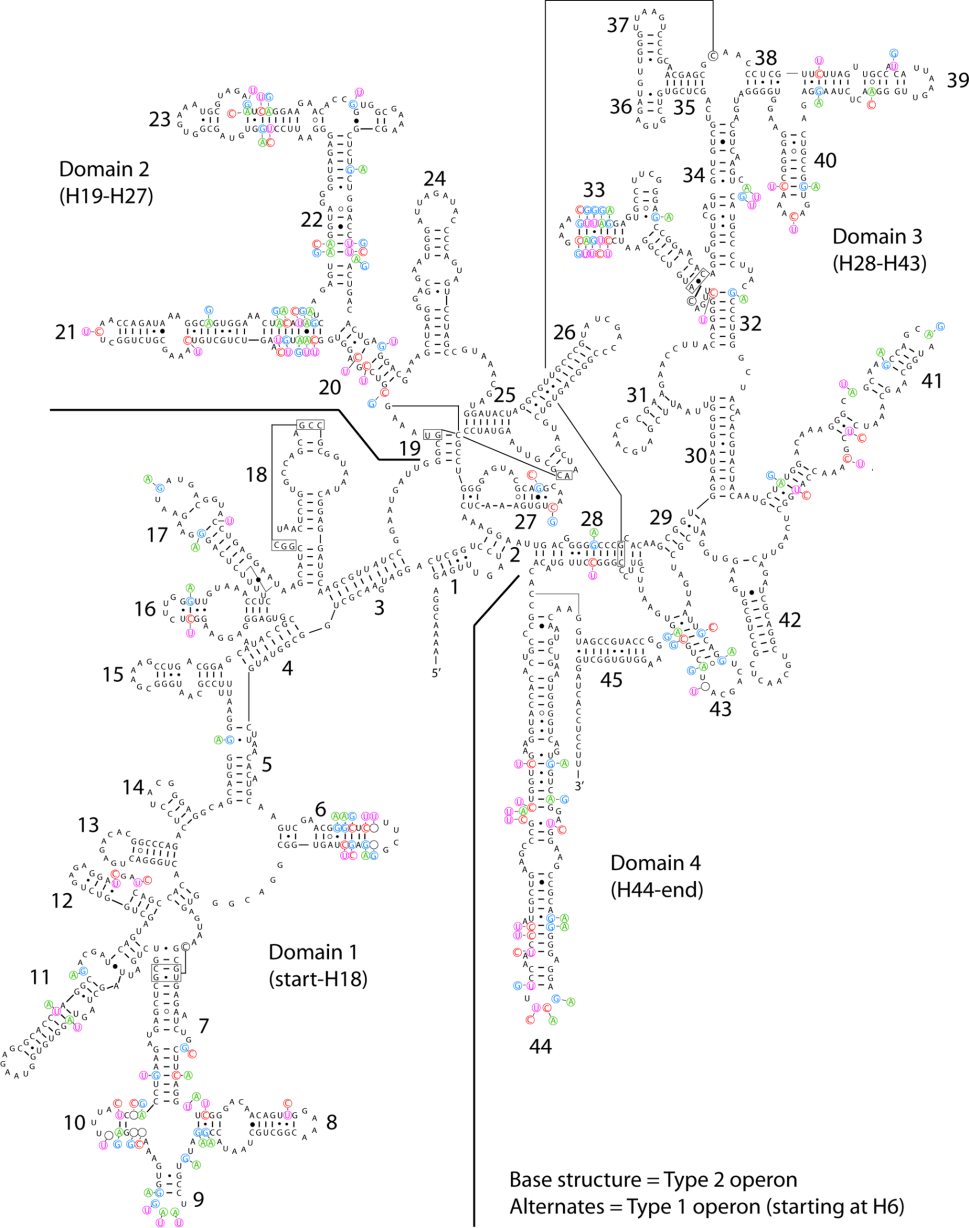
Predicted secondary structure of the complete 16S rRNA molecule for *Scytonema hyalinum* HTT-U-KK4. Type 2 operon is the base structure, and variable bases in the Type 1 operon are shown as alternates. Indels are noted with empty circles where a base was deleted (or an insertion occurred opposite the position), e.g. in H6 and H10. The separation of the four domains are delineated with lines; the end of Domain 2 and beginning of Domain 3 occurs between helices 27 and 28.

While divergent operons in *S*. *hyalinum* were sequenced earlier based on records in NCBI, they went unreported in the literature reporting on the strains in which they were first seen (DC-A in Yaeger et al. [46], HAF2-B2-c1 in Vaccarino et Johansen [47]). Only when intensive efforts in sequencing *Scytonema* strains were undertaken, did recognition that these were highly divergent operons within strains and not an artifact become unavoidable. To determine if the divergent Type 1 operon was a pseudogene, secondary structure of the 16S rRNA molecule was compared within strains. One representative example is shown (strain HTT-U-KK4), and it is clear that the secondary structure was almost perfectly conserved (Fig 2). Only in helices H9 and H10 did a change in structure take place, due to a decoupling of one base pair in the terminal loop of H9, and to four inserted bases in H10. An examination of the locations of the variable bases (which are nearly the same in all strains) shows that the heterogeneity between operons is highly localized in selected helices distributed throughout the 16S rRNA molecule (Fig 2, S3 Table).

**Fig 3:**
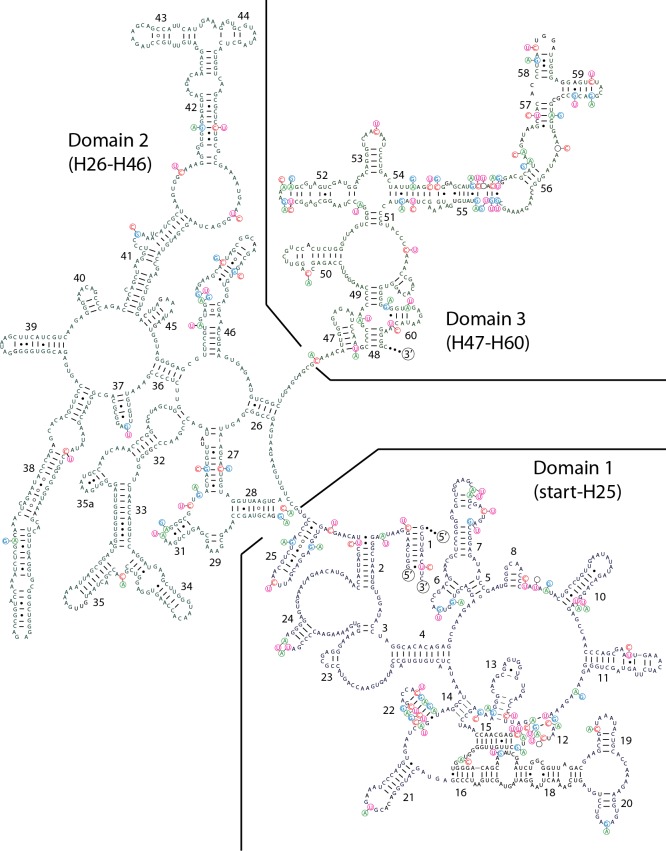
Predicted secondary structure of the complete 5ʹ end of the 23S rRNA molecule for *Scytonema hyalinum* HTT-U-KK4. Type 2 operon is the base structure, and variable bases in the Type 1 operon are shown as alternates. Indels are noted with empty circles where a base was deleted (or an insertion occurred opposite the position). The separation of the first three domains are delineated with lines. Helix 1 consists of the 5ʹ end of the 23S rRNA molecule bound to the 3ʹ end of the molecule, indicated by labels of 5ʹ and 3ʹ. See Fig 4 for the continuation of the structure (i.e., the 3ʹ end).

**Fig 4:**
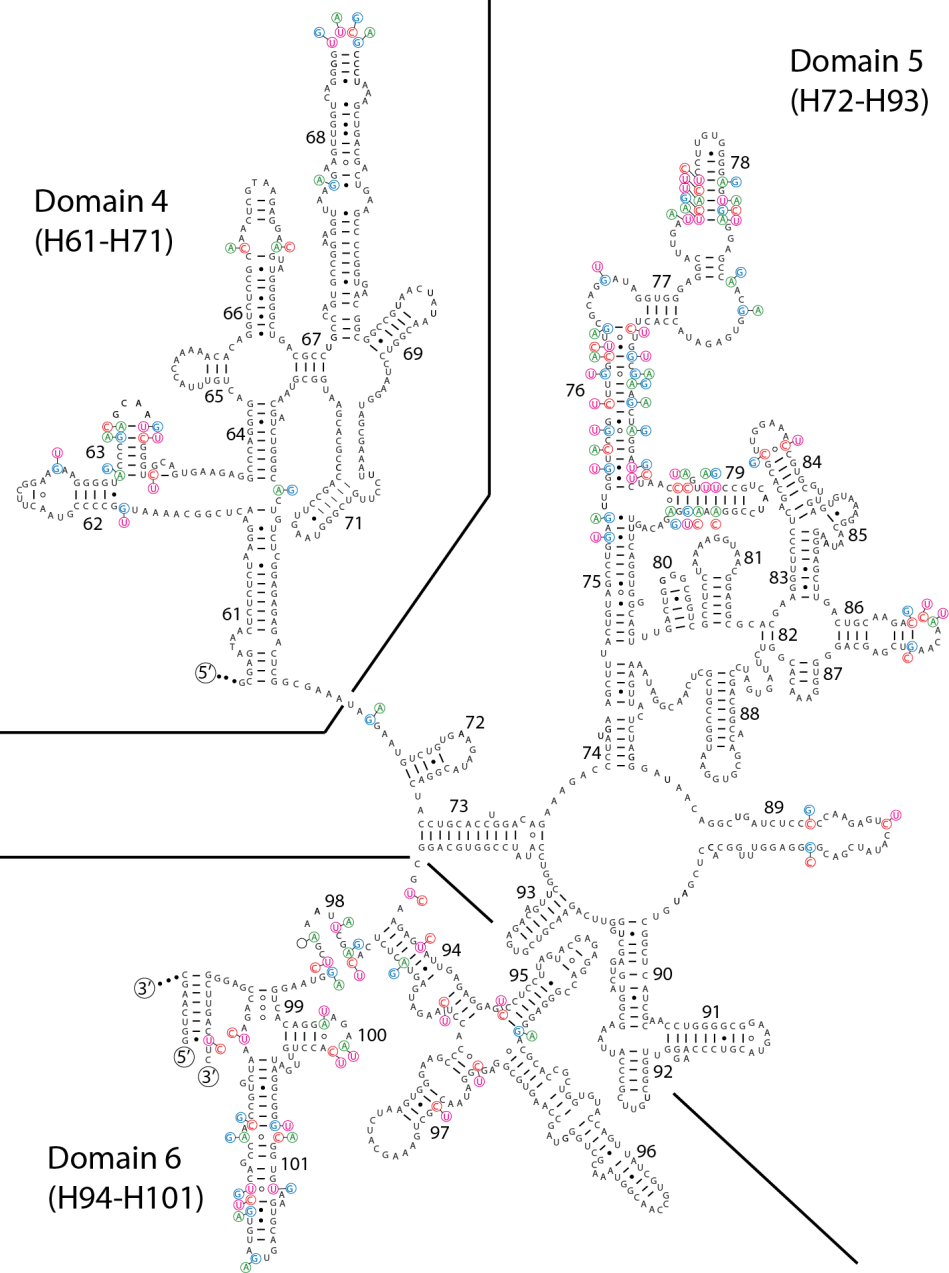
Predicted secondary structure of the complete 3ʹ end of the 23S rRNA molecule for *Scytonema hyalinum* HTT-U-KK4. Type 2 operon is the base structure, and variable bases in the Type 1 operon are shown as alternates. Indels are noted with empty circles where a base was deleted (or an insertion occurred opposite the position). The separation of the last three domains are delineated with lines. Helix 1 consists of the 5ʹ end of the 23S rRNA molecule bound to the 3ʹ end of the molecule, indicated by labels of 5ʹ and 3ʹ. See Fig 3 for the continuation of the structure (i.e., the 5ʹ end).

We recovered the promoter region, leader, 16S rRNA, 16S-23S ITS, 23S rRNA, 23S-5S ITS, 5S rRNA and the terminator region for WJT9-NPBG6B (entire Type 2 operon) and HA4185-MV1 (entire Type 1 operon). A recently available genome of *Scytonema* sequenced by others, *Scytonema* sp. HK-05, also possesses both divergent operons, with the four Type 2 operons in the chromosome and the Type 1 operon on a plasmid. Finally, we sequenced the 23S rRNA gene, 23S-5S ITS, and partial 5S rRNA gene for HTT-U-KK4. All 23S rRNA gene sequences in Type 1 operons for these four strains are highly similar (98.4–99.3%), as are the 23S rRNA sequences for the Type 2 operons (98.5–100%). However, the 23S rRNA genes in the Type 1 and Type 2 operons are much less similar (92.7–93.7%). This level of divergence roughly corresponds to the degree of divergence seen in the 16S rRNA genes. Likewise, the secondary structure of the 23S is preserved for both operons, with highly localized heterogeneity (Figs 3 and 4). Finally, the proximal promoter regions (-10, -35, and -52) match established prokaryotic promoter sequences that bind RNA polymerase holoenzyme containing sigma-70 (Table 1). This combined evidence suggests that both operons are functional and transcribed. However, direct experimental evidence for the transcription of both operons is pending.

**Table 1:** Promoter regions for Type 1 and Type 2 operons. The -10 (Pribnow Box) and -35 promoter regions are considered to be likely functional if 3–6 nucleotides in each match the consensus sequence. The optional -52 promoter may or may not be functional in these promoter regions.

Prokaryotic Consensus	-52		-35		-10		Transcript begins
	AWWWWWTTTTT	. . . .. . . .	TTGACA	. . . .. . . .. . . .. . .. . .	TATAAT	. . . .	. . . .. . .. . ..
Type 2 operons							
NIES 4073 AP018268.1	AAAAATTTTGA	AAAAGGAG	TTGACA	ATGCAGGAGTGGG-TGGA	TATATT	AAAT	AAGTGCCTGAA
NIES 4073 AP018268.1	AAAAATTTTGA	AAAAGGAG	TTGACA	ATGCAGGAGTGGG-TGGA	TATATT	AAAT	AAGTGCCTGAA
NIES 4073 AP018268.1	AAAAAGTTTGA	AATGCCCC	TTGACA	AAAAAAAAAGCGG-TGGC	TAGACT	AGAT	AAA-GTGTGAA
NIES 4073 AP018268.1	AAAAAGTTTGA	AATCCCCC	TTGACA	AAAAAAAAAGCGG-TGGC	TAGACT	AGAT	AAA-GTGTGAA
NIES 4073 AP018268.1	AAAAAGTTTGA	AATGCCCC	TTGACA	AAAAAAAAAGCGG-TGGC	TAGACT	AGAT	AAA-GTGTGAA
HK-05 AP018194.1	AAATTTTTTGA	AAAAGGAG	TTGACA	ATCCAGGAGTGGG-TGGA	TATATT	AAAT	AAGTGCCTGAA
HK-05 AP018194.1	AAATTTTTTGA	AAAAGGAG	TTGACA	ATCCAGGAGTGGG-TGGA	TATATT	AAAT	AAGTGCCTGAA
HK-05 AP018194.1	AAAAAGTTTGA	AAAACCCC	TTGACA	AAAAAAAAAGCGG-TGGC	TAGACT	AGAT	TAA-GTGTGAA
HK-05 AP018194.1	AAAAAGTTTGA	ATTGCCCC	TTGACA	AAAAAAAAAGCGG-TGGC	TAGACT	AGAT	TAA-GTGTGAA
WJT9-NPBG6B KY365452	AATTTTTTTGA	AAAAGGAG	TTGACA	AAAAAAAAAGCGG-TGGC	TATATT	AAAT	AAGTGCCTGAA
HTT-U-KK4 KY365438	. . . .. . .. . ..	. . . .. . . .	. . .. . .	. . . .. . . .. GGA	TATATT	GGAT	AAGTGCCGGAA
Type 1 operons							
HA4185-MV1 KY365490	AAAAGTACGGT	TTCACCTC	CACACA	CTCCCAACGTTGTCTAGT	TACAAT	GAAA	GAGTGTCAAGG
HK-05 Type 1 AP018198.1	AAAAGTACGGT	TTCACCTC	CACACA	CTCCCAATGTTGTCCAGT	TACAAT	GAAA	GAGTGTCAAGG

**Fig 5:**
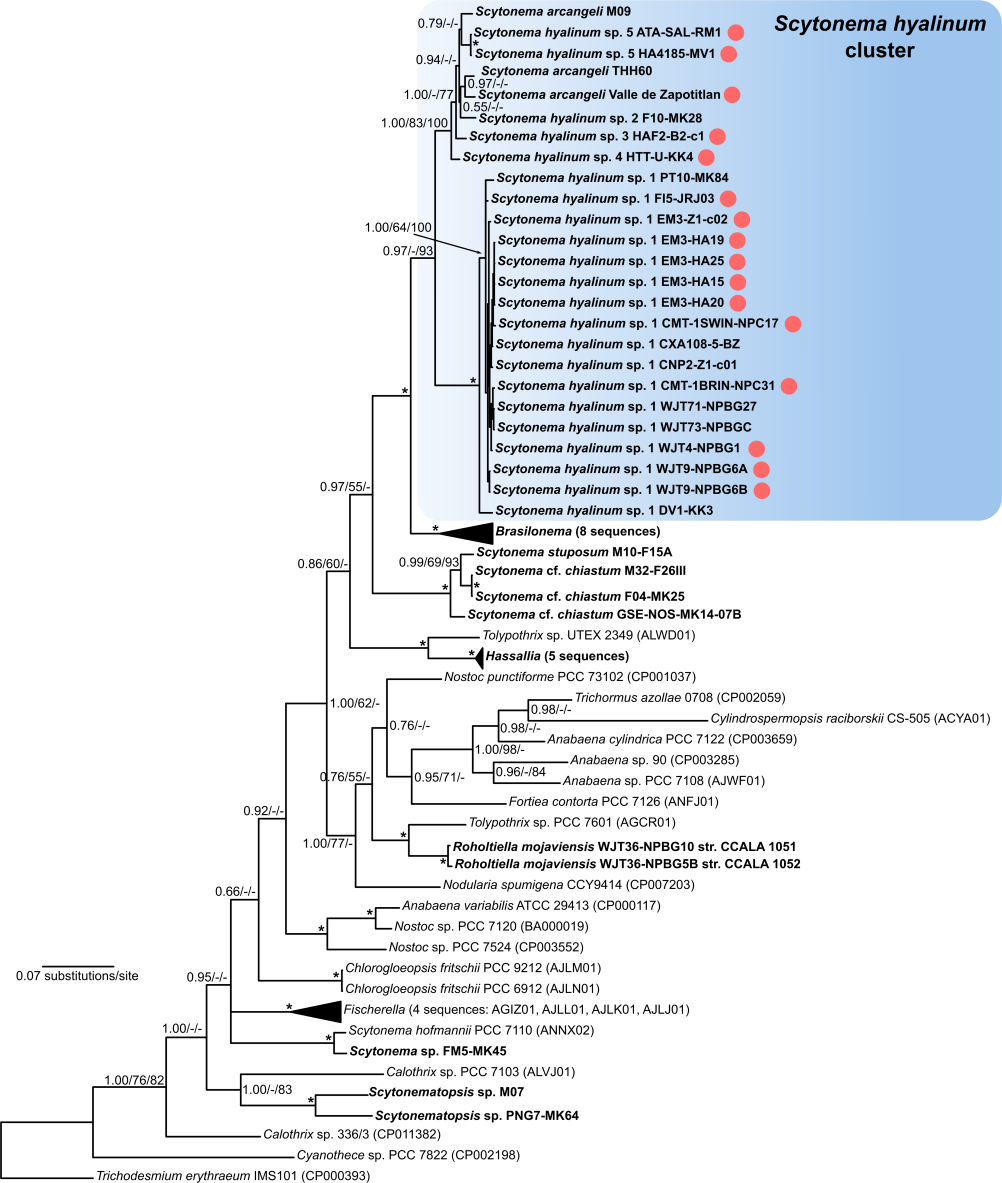
Phylogenetic tree based on multilocus data. The tree was inferred from a concatenated nucleotide alignment of partial *rpo*C1, *rbc*LX, and *nif*D sequences (for details see Materials and methods). The *Scytonema hyalinum* species cluster (shaded box) forms a monophyletic lineage sister to *Brasilonema*. Circles indicate strains in which two divergent (polyphyletic) rRNA operon types were detected. The tree is based on Bayesian Inference; branch supports ≥50% are given at the nodes in this shape: Bayesian Inference/Maximum Likelihood/Neighbor-Joining. Asterisks indicate nodes with 100% support from all methods. Strains sequenced in this study are printed in bold font; accession numbers for the three loci for these strains are listed in S4 Table.

We amplified *rbc*LX, *rpo*C1, and *nif*D, all single copy genes, in numerous *S*. *hyalinum* strains. In no instance did we get a mixed PCR product of any of these genes, indicating a single genome in each strain (i.e. no contaminants). The multilocus phylogeny excluding the 16S rRNA gene showed *Brasilonema* as a sister taxon to *S*. *hyalinum* (Fig 5), a result echoed in the 16S tree, except *Brasilonema* is closest to the Type 1 operon (Fig 1). In examining just the variable positions of the 16S rRNA gene between operons (as shown in Fig 2), we found that *Brasilonema* species had 28% of the positions identical to the Type 1 operon, 54% of the positions identical to the Type 2 operon, and 18% of the positions either unique or a mix of what occurred in the *S*. *hyalinum* operons (S3 Table). Within operon types, bases were very consistent, with only H9 and H33 showing elevated variation among strains within operon type (S3 Table). At present, 23S rRNA sequences are not available for *Brasilonema*.

The presence of the highly divergent Type 1 operon is strongly indicative of HGT. It appears to have come to an ancestor of the *S*. *hyalinum* species complex. At the time of introduction to the ancestral genome, there were likely up to four near-identical Type 2 16S rRNA genes in separate operons, most similar to those in *Brasilonema*, and slightly less similar to those in the type species of *Scytonema* and its sister taxon, *S*. *hofmannii* and *S*. *fritschii*, respectively. The evidence we present indicates that all elements of the Type 1 operon are consistently divergent, suggesting introduction of an entire operon. The localization of the Type 1 operon on a plasmid in HK-05 further supports the HGT hypothesis, as vector-facilitated HGT is more common than direct transfer of chromosomal segments. It is intriguing that this is the first report of an *rrn*-plasmid in cyanobacteria, and only the second report of an *rrn*-plasmid in the bacterial domain [48]. However, there are several unexpected findings associated with this HGT event.

First, we cannot identify the source of the operon. While the Type 1 operon is only slightly more similar to *Brasilonema* species (91.7–92.9%) than to the Type 2 operon of *S*. *hyalinum* (91.0–92.7%), it bears less resemblance to all other cyanobacterial and non-cyanobacterial 16S rRNA sequences. This is consistent with the complexity hypothesis, which posits that HGT of ribosomal genes is very unlikely, but most likely in closely related lineages [49]. The source is likely a member of the Scytonemataceae, but not one that has been sequenced as yet. This raises the inevitable evolutionary question, how did this ancestral taxon become so divergent in the first place, and what selective forces allowed such a deep divergence? HGT is a handy explanation of the Type 1 operon sequence, but it does not address the ultimate origin of the sequence.

Second, we do not understand why gene conversion, a phenomenon observed in other instances of HGT of 16S rRNA genes [13,50], has not been more effective at homogenizing the divergent sequence. There is limited evidence of gene conversion in the *S*. *hyalinum* species complex. In particular, in 14 of the 123 variable positions there were a few reversions among the 21 strains sequenced (S3 Table). However, this is relatively little gene conversion given the fact that the HGT event occurred sufficiently long ago that at least six species have arisen in the lineage since the event occurred.

Third, *all* of the positions variable between the two operon types in *S*. *hyalinum* operons are considered to be among the least conserved in the prokaryotic 16S rRNA molecule according to a recently published summary [51]. This could explain the origin of the divergent sequence in an unknown ancestral heterocytous taxon, but it does not explain the relative invariability in these positions within the Type 1 operon in the *Scytonema hyalinum* species cluster. We conclude that the HGT event, which likely defines the origin of the *Scytonema hyalinum/arcangelii* lineage, is relatively recent in evolutionary terms. It is a challenging notion because “relatively recent” seems inconsistent with a radiation event which has given rise to multiple ecophysiologically and genetically (16S-23S ITS) diverse species. The alternative hypothesis is that the sequence of the Type 1 operon is ancient, but has been tightly constrained by natural selection since its origin.

The persistence of the relatively stable Type 1 operon in this cyanobacterial lineage is evidence that the Type 1 operon imparts some selective advantage. In prokaryotic lineages with macroheterogeneity in ribosomal operons the taxa are often from extreme environments, and adaptive advantage is inferred from this congruence [16,24,52,53]. Condon et al. [54] suggest that additional operons in *E*. *coli* permit more rapid adaptation to changing environmental conditions, and Anda et al. [48] suggest this same adaptability in the ribosomal genes in the high-copy plasmids of *Aureimonas*. *Scytonema hyalinum* is an extremophile, with populations being exposed to rapidly changing temperatures, high conductivities, damaging levels of solar radiation, and rapidly changing moisture conditions. It consequently fits the model expected if adaptive advantage accrues from the additional, divergent operon, especially if it is positioned on a plasmid, as evidenced in the strain HK-05. We cannot hypothesize what advantage the introduced operon imparts, but its widespread presence and sequence stability still suggest adaptive advantage.

## Supporting information

S1 Table Characterization of five distinct operons based on 16S-23S ITS regions recovered from two ribosomal operon types in *Scytonema hyalinum* species cluster. Color coded regions are as follows: leader (blue-block); D1-D1' helix (first green block); D2 (first yellow block); D3 (second yellow block); tRNA-Ile (first red block, when present); V2 helix (second blue block, when present); tRNA-Ala (second red block, when present); Box-B helix (second green block); Box-A (third yellow block); D4 (magenta block); V3 helix (third green block).
(PDF)

S2 Table Summary of p-distance values for 20 *Scytonema* strains for which both operons were observed, and for which long reads were recovered (1170–1485 nucleotides), with comparisons also to eight *Brasilonema* strains. Comparisons between the operons within strain are given in first column (blue highlight). Comparisons among Type 2 operons among the 20 strains are given in the first block (yellow highlight), followed by the comparisons among Type 1 operons (green highlight). The third block gives comparisons among *Brasilonema* spp. and the Type 2 operon (yellow highlight), followed by comparisons among *Brasilonema* spp. and the Type 1 operon (green highlight).
(PDF)

S3 Table Alignment of bases consistently variable between operons in *Scytonema hyalinum*. Color coding: yellow = consensus bases consistent with Type 2 operons; blue = consensus bases consistent with Type 1 operons; green = consensus bases unique to *Brasilonema*; gray = variable bases not consistent with any consensus base. Question marks = missing data. Helix number given in accordance to Fig 2, with position numbers based on 1501 positions in alignment of complete 16S rRNA molecule in *Scytonema hyalinum* sensu lato. *Brasilonema* is 54% like Type 2 operons, 28% like Type 1 operons, and 19% unique to *Brasilonema* or variable (consensus with < 85% saturation).
(PDF)

S4 Table Summary of NCBI accession numbers for *rbc*LX, *rpo*C1, and *nif*D nucleotide sequences obtained in this study. (PDF)
