# Supplementary material for: Highly divergent 16S rRNA sequences in ribosomal operons of Scytonema hyalinum (Cyanobacteria)
Source: PLoS One. 2017 Oct 26;12(10):e0186393. doi: 10.1371/journal.pone.0186393 (PMC5658200; doi:10.1371/journal.pone.0186393)

S1 Table. Characterization of five distinct operons based on 16S-23S ITS regions recovered from two ribosomal operon types in *Scytonema hyalinum* species cluster. Color coded regions are labeled at the top of each block of each operon type. For the helices, the basal clamps regions (5' and 3' ends that define the base of the helix) are in green shading, for ease in seeing differences in the operons.

Type 1 operon: Attached to Type 1 16S rRNA gene; distinguished by distinct leader (AACAGGGA, compared to TNTAGGGA); lacks both tRNA genes; D3 is GGTAY (compared to GGTTC in all Type 2 operons); D4 starts ATTG (compared to ACTG in all Type 2 operons).

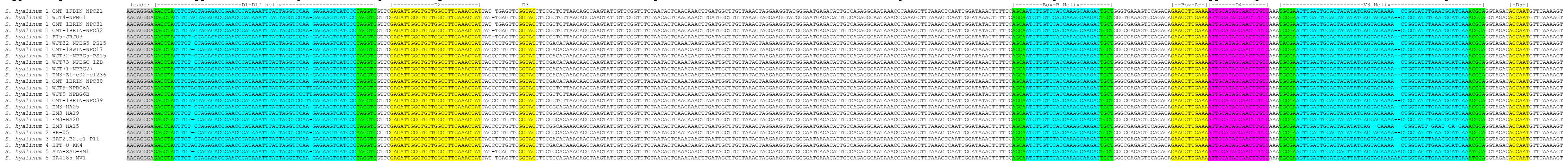

Type 2A operon: Attached to Type 2 16S rRNA gene; distinguished from other Type 2 operons by absence of both tRNA genes and shortness of D1-D1' (maximally 68 nucleotides long).

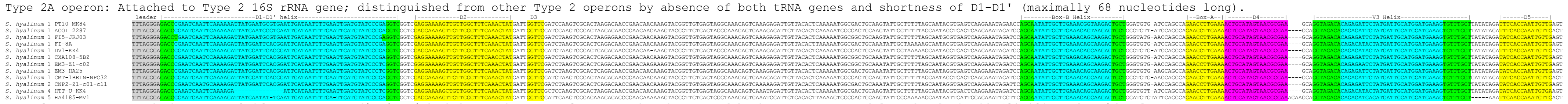

Type 2B Operon: Attached to Type 2 16S rRNA gene; distinguished from other Type 2 operons by absence of both tRNA genes and length of D1-D1' (81-101 nucleotides long).

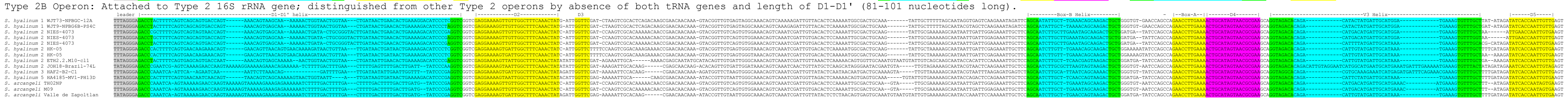

Type 2C operon: Attached to Type 2 16S rRNA gene; distinguished from other Type 2 operons by presence of both tRNA genes and length of D1-D1' (80-90 nucleotides long).

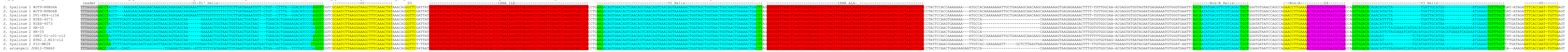

Type 2D Operon: Attached to Type 2 16S rRNA gene; distinguished from other Type 2 operons by presence of both tRNA genes, and different from DV1-KK4 Type 2C operon in sequence and length of D1-D1' helix.

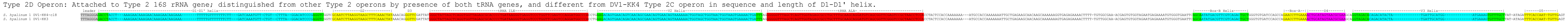

Supplement: S1 Table — Color coded regions are as follows: leader (blue-block); D1-D1' helix (first green block); D2 (first yellow block); D3 (second yellow block); tRNA-Ile (first red block, when present); V2 helix (second blue block, when present); tRNA-Ala (second red block, when present); Box-B helix (second green block); Box-A (third yellow block); D4 (magenta block); V3 helix (third green block). (PDF) [file pone.0186393.s001.pdf]
