# Supplementary material for: Highly divergent 16S rRNA sequences in ribosomal operons of Scytonema hyalinum (Cyanobacteria)
Source: PLoS One. 2017 Oct 26;12(10):e0186393. doi: 10.1371/journal.pone.0186393 (PMC5658200; doi:10.1371/journal.pone.0186393)
Supplement: S2 Table — Comparisons between the operons within strain are given in first column (blue highlight). Comparisons among Type 2 operons among the 20 strains are given in the first block (yellow highlight), followed by the comparisons among Type 1 operons (green highlight). The third block gives comparisons among Brasilonema spp. and the Type 2 operon (yellow highlight), followed by comparisons among Brasilonema spp. and the Type 1 operon (green highlight). (PDF) [file pone.0186393.s002.pdf]

S2 Table. Summary of p-distance values for 20 *Scytonema* strains for which both operons were observed, and for which long reads were recovered (1170-1485 nucleotides), with comparisons also to eight *Brasilonema* strains.

Comparisons between the operons *within strain* are given in first column (blue highlight). Comparisons among Type 2 operons among the 20 strains are given in the first block (yellow highlight), followed by the comparisons among Type 1 operons (green highlight). The third block gives comparisons among *Brasilonema* spp. and the Type 2 operon (yellow highlight), followed by comparisons among *Brasilonema* spp. and the Type 1 operon (green highlight).

T1/T2		1	2	3	4	5	6	7	8	9	10	11	12	13	14	15	16	17	18	19	20
0.08360	1 *Scytonema hyalinum*_Sp1 CMT-1BRIN-NPC30	-																			
0.08042	2 *Scytonema hyalinum*_Sp1 CMT-1BRIN-NPC31	0.00000	-																		
0.08453	3 *Scytonema hyalinum*_Sp1 CMT-1BRIN-NPC32	0.00078	0.00078	-																	
0.08269	4 *Scytonema hyalinum*_Sp1 CMT-1BRIN-NPC39	0.00000	0.00000	0.00078	-																
0.07673	5 *Scytonema hyalinum*_Sp1 CMT-1SWIN-NPC17	0.00081	0.00081	0.00161	0.00081	-															
0.07498	6 *Scytonema hyalinum*_Sp1 EM3-HA15	0.00000	0.00000	0.00078	0.00000	0.00081	-														
0.07498	7 *Scytonema hyalinum*_Sp1 EM3-HA19	0.00000	0.00000	0.00078	0.00000	0.00081	0.00000	-													
0.07425	8 *Scytonema hyalinum*_Sp1 EM3-HA20	0.00000	0.00000	0.00078	0.00000	0.00081	0.00000	0.00000	-												
0.07892	9 *Scytonema hyalinum*_Sp1 EM3-HA25	0.00000	0.00000	0.00071	0.00000	0.00081	0.00000	0.00000	0.00000	-											
0.08049	10 *Scytonema hyalinum*_Sp1 EM3-Z1-c02	0.00000	0.00000	0.00070	0.00000	0.00081	0.00000	0.00000	0.00000	0.00000	-										
0.08190	11 *Scytonema hyalinum*_Sp1 FI5-JRJ03	0.00000	0.00000	0.00071	0.00000	0.00081	0.00000	0.00000	0.00000	0.00000	0.00000	-									
0.07893	12 *Scytonema hyalinum*_Sp1 WJT4-NPBG1	0.00000	0.00000	0.00078	0.00000	0.00081	0.00000	0.00000	0.00000	0.00000	0.00000	0.00000	-								
0.08545	13 *Scytonema hyalinum*_Sp1 WJT9-NPBG6A	0.00320	0.00320	0.00353	0.00321	0.00410	0.00320	0.00320	0.00320	0.00282	0.00282	0.00282	0.00321	-							
0.08474	14 *Scytonema hyalinum*_Sp1 WJT9-NPBG6B	0.00241	0.00241	0.00283	0.00241	0.00328	0.00241	0.00241	0.00241	0.00211	0.00212	0.00212	0.00241	0.00071	-						
0.07984	15 *Scytonema arcangelii* FL17-MK96	0.00954	0.00955	0.01033	0.00955	0.01058	0.00954	0.00954	0.00954	0.00955	0.00955	0.00954	0.00878	0.01200	0.01120	-					
0.07997	16 *Scytonema arcangelii* Valle de Zapotlan	0.00952	0.00953	0.01268	0.00953	0.01056	0.00952	0.00952	0.00952	0.01197	0.01197	0.01196	0.00876	0.01412	0.01341	0.00000	-				
0.08544	17 *Scytonema hyalinum*_Sp3 HAF2-B2-C1	0.01434	0.01434	0.01340	0.01435	0.01550	0.01434	0.01434	0.01434	0.01269	0.01269	0.01269	0.01357	0.01059	0.00988	0.00639	0.00917	-			
0.08421	18 *Scytonema hyalinum*_Sp4 HTT-U-KK4	0.00953	0.00953	0.00916	0.00875	0.01057	0.00953	0.00953	0.00953	0.00845	0.00842	0.00845	0.00876	0.00918	0.00848	0.00316	0.00634	0.00565	-		
0.08966	19 *Scytonema hyalinum*_Sp5 HA4185-MV1	0.01354	0.01355	0.01340	0.01278	0.01467	0.01354	0.01354	0.01355	0.01268	0.01268	0.01268	0.01278	0.01341	0.01270	0.00717	0.01057	0.00564	0.00423	-	
0.08702	20 *Scytonema hyalinum*_Sp5 ATA-SAL-RM1	0.01433	0.01434	0.01512	0.01355	0.01548	0.01433	0.01433	0.01354	0.01433	0.01433	0.01433	0.01357	0.01519	0.01440	0.00796	0.00793	0.00634	0.00478	0.00080	-
	1 *Scytonema hyalinum*_Sp1 CMT-1BRIN-NPC30	-																			
	2 *Scytonema hyalinum*_Sp1 CMT-1BRIN-NPC31	0.00843	-																		
	3 *Scytonema hyalinum*_Sp1 CMT-1BRIN-NPC32	0.00773	0.00773	-																	
	4 *Scytonema hyalinum*_Sp1 CMT-1BRIN-NPC39	0.01123	0.00491	0.01120	-																
	5 *Scytonema hyalinum*_Sp1 CMT-1SWIN-NPC17	0.00492	0.00352	0.00421	0.00703	-															
	6 *Scytonema hyalinum*_Sp1 EM3-HA15	0.00984	0.00563	0.01054	0.00913	0.00774	-														
	7 *Scytonema hyalinum*_Sp1 EM3-HA19	0.00984	0.00563	0.01054	0.00913	0.00774	0.00000	-													
	8 *Scytonema hyalinum*_Sp1 EM3-HA20	0.00984	0.00563	0.01054	0.00913	0.00774	0.00000	0.00000	-												
	9 *Scytonema hyalinum*_Sp1 EM3-HA25	0.00983	0.00562	0.01048	0.00911	0.00774	0.00000	0.00000	0.00000	-											
	10 *Scytonema hyalinum*_Sp1 EM3-Z1-c02	0.00913	0.00352	0.00843	0.00703	0.00563	0.00632	0.00632	0.00632	0.00632	-										
	11 *Scytonema hyalinum*_Sp1 FI5-JRJ03	0.00773	0.00070	0.00702	0.00421	0.00282	0.00492	0.00492	0.00492	0.00492	0.00281	-									
	12 *Scytonema hyalinum*_Sp1 WJT4-NPBG1	0.00492	0.00633	0.00559	0.00981	0.00281	0.00913	0.00913	0.00913	0.00909	0.00422	0.00563	-								
	13 *Scytonema hyalinum*_Sp1 WJT9-NPBG6A	0.01053	0.00421	0.01050	0.00070	0.00633	0.00843	0.00843	0.00843	0.00840	0.00632	0.00351	0.00911	-							
	14 *Scytonema hyalinum*_Sp1 WJT9-NPBG6B	0.01053	0.00421	0.01050	0.00070	0.00633	0.00843	0.00843	0.00843	0.00840	0.00632	0.00351	0.00911	0.00000	-						
	15 *Scytonema arcangelii* FL17-MK96	0.01262	0.01263	0.01423	0.01660	0.01180	0.00948	0.00948	0.00948	0.00950	0.01188	0.01183	0.01183	0.01580	0.01580	-					
	16 *Scytonema arcangelii* Valle de Zapotlan	0.01262	0.01263	0.01423	0.01660	0.01180	0.00948	0.00948	0.00948	0.00950	0.01188	0.01183	0.01183	0.01580	0.01580	0.00000	-				
	17 *Scytonema hyalinum*_Sp3 HAF2-B2-C1	0.00914	0.00352	0.00843	0.00703	0.00563	0.00211	0.00211	0.00211	0.00211	0.00422	0.00281	0.00703	0.00632	0.00632	0.00870	0.00870	-			
	18 *Scytonema hyalinum*_Sp4 HTT-U-KK4	0.00983	0.00421	0.00908	0.00770	0.00633	0.00281	0.00281	0.00281	0.00279	0.00351	0.00351	0.00769	0.00700	0.00700	0.00792	0.00792	0.00070	-		
	19 *Scytonema hyalinum*_Sp5 HA4185-MV1	0.01476	0.01828	0.01757	0.01615	0.01477	0.01685	0.01685	0.01685	0.01687	0.01897	0.01757	0.01475	0.01545	0.01545	0.01181	0.01181	0.01615	0.01685	-	
	20 *Scytonema hyalinum*_Sp5 ATA-SAL-RM1	0.01480	0.01833	0.01761	0.01619	0.01479	0.01690	0.01690	0.01690	0.01691	0.01902	0.01761	0.01479	0.01549	0.01549	0.01184	0.01184	0.01619	0.01690	0.00000	-
	Brasilonema BZ-HDL-007 vs Type 2 operon	0.04850	0.04854	0.04719	0.04857	0.05050	0.04850	0.04850	0.04851	0.04650	0.04647	0.04646	0.04868	0.04944	0.04873	0.05250	0.05276	0.05149	0.05073	0.05359	0.05657
	Brasilonema CR6-4B vs Type 2 operon	0.04873	0.04873	0.04669	0.04876	0.05071	0.04873	0.04873	0.04874	0.04596	0.04596	0.04596	0.04875	0.04457	0.04386	0.05193	0.05228	0.04596	0.04811	0.05091	0.05430
	Brasilonema CR6-5A vs Type 2 operon	0.05006	0.05011	0.04787	0.04936	0.05211	0.05006	0.05006	0.05008	0.04719	0.04701	0.04715	0.05025	0.04943	0.04872	0.05404	0.05415	0.05148	0.05109	0.05216	0.05574
	Brasilonema KEN-MK50 vs Type 2 operon	0.04846	0.04850	0.04646	0.04854	0.05047	0.04846	0.04846	0.04847	0.04578	0.04564	0.04574	0.04864	0.04871	0.04800	0.05244	0.05204	0.05077	0.05183	0.05146	0.05492
	Brasilonema M31-FB20B vs Type 2 operon	0.05404	0.05408	0.04930	0.05411	0.05619	0.05404	0.05404	0.05405	0.05001	0.04986	0.04997	0.05424	0.05296	0.05225	0.05639	0.05345	0.05360	0.05464	0.05498	0.05966
	Brasilonema P09-MK13 vs Type 2 operon	0.05636	0.05641	0.05281	0.05645	0.05857	0.05636	0.05636	0.05638	0.05212	0.05209	0.05208	0.05657	0.05296	0.05225	0.05872	0.05556	0.05359	0.05354	0.05568	0.06040
	Brasilonema PT5-MK70 vs Type 2 operon	0.06050	0.06054	0.05566	0.06058	0.06280	0.06050	0.06050	0.06052	0.05496	0.05493	0.05492	0.06072	0.05581	0.05510	0.06128	0.05840	0.05644	0.05640	0.05853	0.06457
	Brasilonema TH04-EMA vs Type 2 operon	0.05467	0.05472	0.04999	0.05476	0.05685	0.05467	0.05467	0.05469	0.05070	0.05055	0.05066	0.05487	0.05224	0.05153	0.05624	0.05345	0.05287	0.05396	0.05497	0.05950
	Brasilonema BZ-HDL-007 vs Type 1 operon	0.08033	0.07751	0.08156	0.07870	0.07691	0.07543	0.07543	0.07543	0.07594	0.07756	0.07614	0.08015	0.07800	0.07800	0.06784	0.06784	0.07473	0.07452	0.07614	0.07629
	Brasilonema CR6-4B vs Type 1 operon	0.07988	0.07494	0.08126	0.07629	0.07704	0.07351	0.07351	0.07351	0.07351	0.07564	0.07422	0.07985	0.07559	0.07559	0.06881	0.06881	0.07280	0.07209	0.07561	0.07570
	Brasilonema CR6-5A vs Type 1 operon	0.08245	0.07752	0.08339	0.07872	0.07904	0.07545	0.07545	0.07545	0.07573	0.07757	0.07616	0.08181	0.07801	0.07801	0.07105	0.07105	0.07475	0.07432	0.07615	0.07629
	Brasilonema KEN-MK50 vs Type 1 operon	0.08105	0.07613	0.08206	0.07732	0.07763	0.07405	0.07405	0.07405	0.07440	0.07617	0.07476	0.08042	0.07662	0.07662	0.06866	0.06866	0.07335	0.07299	0.07686	0.07701
	Brasilonema M31-FB20B vs Type 1 operon	0.07542	0.07542	0.07504	0.07662	0.07199	0.07335	0.07335	0.07335	0.07369	0.07687	0.07406	0.07483	0.07591	0.07591	0.06710	0.06710	0.07265	0.07368	0.07122	0.07136
	Brasilonema P09-MK13 vs Type 1 operon	0.07823	0.07823	0.07804	0.07871	0.07481	0.07757	0.07757	0.07757	0.07806	0.07969	0.07687	0.07805	0.07801	0.07801	0.07180	0.07180	0.07687	0.07805	0.07262	0.07276
	Brasilonema PT5-MK70 vs Type 1 operon	0.07262	0.07333	0.07386	0.07382	0.06919	0.07267	0.07267	0.07267	0.07318	0.07480	0.07197	0.07245	0.07311	0.07311	0.06797	0.06797	0.07197	0.07317	0.06701	0.06714
	Brasilonema TH04-EMA vs Type 1 operon	0.07612	0.07612	0.07574	0.07731	0.07269	0.07545	0.07545	0.07545	0.07579	0.07757	0.07476	0.07552	0.07661	0.07661	0.06937	0.06937	0.07475	0.07578	0.07121	0.07135
