# Supplementary material for: Highly divergent 16S rRNA sequences in ribosomal operons of Scytonema hyalinum (Cyanobacteria)
Source: PLoS One. 2017 Oct 26;12(10):e0186393. doi: 10.1371/journal.pone.0186393 (PMC5658200; doi:10.1371/journal.pone.0186393)
Supplement: S3 Table — Color coding: yellow = consensus bases consistent with Type 2 operons; blue = consensus bases consistent with Type 1 operons; green = consensus bases unique to Brasilonema; gray = variable bases not consistent with any consensus base. Question marks = missing data. Helix number given in accordance to Fig 2, with position numbers based on 1501 positions in alignment of complete 16S rRNA molecule in Scytonema hyalinum sensu lato. Brasilonema is 54% like Type 2 operons, 28% like Type 1 operons, and 19% unique to Brasilonema or variable (consensus with < 85% saturation). (PDF) [file pone.0186393.s003.pdf]

S3 Table. Alignment of bases consistently variable between operons in *Scytonema hyalinum*. Color coding: yellow = consensus bases consistent with Type 2 operons; blue = consensus bases consistent with Type 1 operons; green = consensus bases unique to *Brasilonema*; gray = variable bases not consistent with any consensus base. Question marks = missing data. Helix number given in accordance to Fig. 2, with position numbers based on 1501 positions in alignment of complete 16S rRNA molecule in *Scytonema hyalinum sensu lato*. *Brasilonema* is 54% like Type 2 operons, 28% like Type 1 operons, and 19% unique to *Brasilonema* or variable (consensus with < 85% saturation).

Helix	HHHHHHHHHHHHH HHHHH HHHHHHHHHHHHHHHHHHHHHHHHHHHHHHHHHHHHHHHHHHHHHHHHHHHHHHHHHHHHHHH HHHHHHHHHHHHHHHHHHHHHHHHH<br>0000000000000 00000 00001111110111110111112222222222222222222222222222222222222223333333333333333444433444 4444442444444444444444<br>6666666666677 88888 99990000000711122566777000111111111111223333333222220778233333333333999900042111 3333338444444444444444
Nucleotide position	00000000000000000000000000000000000000000000000000000000000000000000111111111111111111111111111111111111<br>000000000011111111111111111112222233344455555555555566666666666666777888999999999999001111112222233333333333344444444444<br>67777778881222446667777888899924578399224233444445789900011335566799001558466667777797800222570349901222247888900011122334<br>9012357802393791812512341245248772814291919352346793889123124589124190131634234612345045190487109912931267572349027012 01260
Scytonema hyalinum sp. 1 CMT-1BRIN-NPC30	GA--CCTCGGTGCTCTGGGGTATG--A-TAGATGTTGCGGGCCCCCAAGTCCAACTAGAATGGACAGGTTAGGCGCCTCTGGGAGGCTAGGCCAGATTTCGG-ACA?????????????????
Scytonema hyalinum sp. 1 CMT-1BRIN-NPC31	GA--CCTCGGTGCTCTGGGGTATG--A-TAGATGTTGCGGGCCCCCAAGTCCAACTAGAATGGACAGGTTAGGCGCCTCTGGGAGGCTAGGCCAGATTTCGG-ACA?????????????????
Scytonema hyalinum sp. 1 CMT-1BRIN-NPC32	GA--CCTCGGTGCTCTGGGGTATG--A-TAGATGTTGCGGGCCCCCAAGTCCAACTAGAATGGACAGGTTAGGCGCCTCTGGGAGACTAGGCCAGATTTCGG-ACACCCACCCTTTCGGGTAG
Scytonema hyalinum sp. 1 CMT-1BRIN-NPC39	GA--CCTCGGTGCTCTGGGGTATG--A-TAGATGTTGCGGGCCCCCAAGTCCAACTAGAATGGACAGGTTAGGCGCCTCTGGGAGGCTAGGCCAGATTTCGG-ACA?????????????????
Scytonema hyalinum sp. 1 CMT-1SWIN-NPC17	GA--CCTCGGTGCTCTGGGGTATG--A-TAGATGTTGCGGGCCCCCAAGTCCAACTAGAATGGACAGGTTAGGCGYCTCTGGGAGGCTAGGCCAGATTTC???????????????????????
Scytonema hyalinum sp. 1 CNP2-Z1-C01	????????????????????????????????????GCGGGCCCCCAAGTCCAACTAGAATGGACAGGTTAGGCGCCTCTGGGAGGCTAGGCCAGATTTCGG-ACACCCACCCTTTCGGGTAG
Scytonema hyalinum sp. 1 DV1-KK3	?A--CCTCGGTGCTCTGGGGTTGG--A-TAGATGTTGCGGGCCCCCAAGTCCAACTAGAATGGACAGGTTAGGCGCCTCTGGGAGGCTAGGCCAGATTTCGG-ACACCCACCCTTTCGGGTAG
Scytonema hyalinum sp. 1 DV1-KK4	GA--CCTCGGTGCTCTGGGGTTGG--A-TAGATGTTGCGGGCCCCCAAGTCCAACTAGAATGGACAGGTTAGGCGCCTCTGGGAGGCTAGGCCAGATTTCGG-ACACCCACCCTTTCGGGTAG
Scytonema hyalinum sp. 1 EM3-HA15	GA--CCTCGGTGCTCTGGGGTATG--A-TAGATGTTGCGGGCCCCCAAGTCCAACTAGAATGGACAGGTTAGGCGCCTCTGGGAGGCTAGGCCAGATTTCGG-ACA?????????????????
Scytonema hyalinum sp. 1 EM3-HA19	GA--CCTCGGTGCTCTGGGGTATG--A-TAGATGTTGCGGGCCCCCAAGTCCAACTAGAATGGACAGGTTAGGCGCCTCTGGGAGGCTAGGCCAGATTTCGG-ACA?????????????????
Scytonema hyalinum sp. 1 EM3-HA20	GA--CCTCGGTGCTCTGGGGTATR--A-TAGATGTTGCGGGCCCCCAAGTCCAACTAGAATGGACAGGTTAGGCGCCTCTGGGAGGCTAGGCCAGATTTCGG-ACA?????????????????
Scytonema hyalinum sp. 1 EM3-HA25	GA--CCTCGGTGCTCTGGGGTATG--A-TAGATGTTGCGGGCCCCCAAGTCCAACTAGAATGGACAGGTTAGGCGCCTCTGGGAGGCTAGGCCAGATTTCGG-ACACCCACCCTTTCGGGTAG
Scytonema hyalinum sp. 1 EM3-Z1_c02	GA--CCTCGGTGCTCTGGGGTATG--A-TAGATGTTGCGGGCCCCCAAGTCCAACTAGAATGGACAGGTTAGGCGCCTCTGGGAGGCTAGGCCAGATTTCGG-ACACCCACCCTTTCGGGTAG
Scytonema hyalinum sp. 1 FI5-JRJ03_	GA--CCTCGGTGCTCTGGGGTATG--A-TAGATGTTGCGGGCCCCCAAGTCCAACTAGAATGGACAGGTTAGGCGCCTCTGGGAGGCTAGGCCAGATTTCGG-ACACCCACCCTTTCGGGTAG
Scytonema_hyalinum sp. 1 FI8A-2-AF334699	????????????????????????????????????GCGGGCCCCCAAGTCCAACTAGAATGGACAGGTTAGGCGCCTCTGGGAGGCTAGGCCAGATTTCGG-ACACCCACCCTTTCGGGTAG
Scytonema hyalinum sp. 1 WJT4-NPBG1	?????CTCGGTGCTCTGGGGTATG--A-TAGATGTTGCGGGCCCCCAAGTCCAACTAGAATGGACAGGTTAGGCGCCTCTGGGAGGCTAGGCCAGATTTCGG-ACA?????????????????
Scytonema hyalinum sp. 1 WJT9-NPBG6A	???????????GCTCTGGGGTTGG--A-TAGATGTTGCGGGCCCCCAAGTCCAACTAGAATGGACAGGTTAGGCGCCTCTGGGAGGCTAGGCCAGATTTCGG-ACACCCACCCTTTCGGGTAG
Scytonema hyalinum sp. 1 WJT9-NPBG6B	???????????GCTCTGGGGTTGG--A-TAGATGTTGCGGGCCCCCAAGTCCAACTAGAATGGACAGGTTAGGCGCCTCTGGGAGGCTAGGCCAGATTTCGG-ACACCCACCCTTTCGGGTAG
Scytonema hyalinum sp. 1 PT10-MK84	?G--CCTCGGTGCTCTGGGGTTGG--A-TAGATGTTGCGGGCCCCCAAGTCCAACTAGAATGGACAGGTTAGGCGCCTCTGGGAGGCTAGGCCAGATTTCGG-ACACCCACCCTTTCGGGTAG
Scytonema hyalinum sp. 1 ACOI2287	GG--CCTCGGTGCTCTGGGGTATG--A-TAGATGTTGCGGGCCCCCAAGTCCAACTAGAATGGACAGGTTAGGCGCCTCTGGGAGGCTAGGCCAGATTTCGG-ACACCCACCCTTTCGGGTAG
Scytonema hyalinum sp. 2 JOH18	GG--CCTAGGCGCTCTGGGGGTGG--A-TAGATGTCGCGGTCCCCCAAGACCAACTAGAATGGACAGGTTAGGCGCCTCTGGGGGGCTAGGCCAGATTGTGG-ACACCCACCCTTTCGGGTAG
Scytonema hyalinum sp. 2 F10-MK28	GA--CCTCGGTGCTCTGGGGGTGG--A-TAGATGTTGCGGACCCCCAAGACCAACTAGAATGGACAGGTTAGGCGCCTCTGGGAGGCTAGGCCAGATTGTGG-ACACCCACCCTCTCGGGTAG
Scytonema hyalinum sp. 2 ETH2.2.M10	GG--CCTCGGCGCTCTGGGGAAGG--A-TAGATGTTGCGGTCCCCCAAGTCCAACTAGAATGGACAGGTAAGGCGCTTCTGGGAGGCTAGGCCAGATTGTGG-ACACCCACCCTTTCGGGTAG
Scytonema_hyalinum sp. 2 HK-05	GG--CCTCGGCGCTCTGGGGTTTG--A-TAGATGTTGCGGGCCCCCAAGTCCAACTAGAATGGACAGGTAAGGCGCCTCTGAGAGGCGCGGCCAGATTGTGG-ACACCCACCCTCTCGGGTAG
Scytonema hyalinum sp. 3 HAF2-B2-C1	?G--CCTCGGCGCTCTGGGGTTGG--A-TAGATGTTGCGGACCCCCAAGTCCAACTAGAATGGACAGGTTAGGCGCCTGCGTTAGGCTAGGCCAGATTGTGG-ACACCCACCCTTTCGGGTAG
Scytonema hyalinum sp. 4 HTT-U-KK4	GG--CCTCGGCGCTCTGGGGAAGG--A-TAGATGTTGCGGGCCCCCAAGTCCAACTAGAATGGACAGGTTAGGCGCCTGCGTTAGGCTAGGCCAGATTGTGG-ACACCCACCCTTTCGGGTAG
Scytonema hyalinum sp. 5 HA4185-MV1	GG--CCTCGGCGCTCTGGGGAAGG--A-TAGATGTTGCGGGCCCCCAAGTCCAACTAGAATGGACAGGTAAGGCGCCTGCGTTAGGCTAGGCCAGATTGTGG-ACACCCACCCTTTCGGGTAG
Scytonema hyalinum sp. 5 ATA-SAL-RM1	???????CGGCGCTCTGGGGAAGA--A-TAGATGTTGCGGGCCCCCAAGTCCAACTAGAATGGACAGGTAAGGCGCCTGCGTTAGGCTAGGCCAGATTGTGG-ACA?????????????????
Scytonema_arcangeli CCIBt3134	????????????????????????????????????GCGGACCCCCAAGTCCAACTAGAATGGACAGGTTAGGCGCCTGCGTTAGGCTAGGCCAGATTGTGG-ACACCCACCCTTTCGGGTAG
Scytonema arcangeli JOH12	GG--CCTCGGCGCTCTGGGGTTGG--A-TAGATGTTGCGGACCCCCAAGTCCAACTAGAATGGACAGGTTAGGCGCCTGCGTTAGGCTAGGCCAGATTGTGG-ACACCCACCCTTTCGGGTAG
Scytonema_arcangeli Valle de Zapoltlan	GG--CCTCGGCGCTCTGGGGTATG--A-TAGATGTTGCGGACCCCCAAGTCCAACTAGAATGGACAGGTTAGGCGCCTGCGTTAGGCTAGGCCAGATTGTGG-ACACCCACCCTCTCGGGTAG
Scytonema arcangeli FL17-MK96	GG--CCTCGGCGCTCTGGGGTATG--A-TAGATGTTGCGGACCCCCAATTCCAACTAGAATGGACAGGTTAGGCGCCTGCGTTAGGCTAGGCCAGATTGTGG-ACA?????????????????
Scytonema arcangeli TH02-NP	GG--CCTCGGCGCTCTGGGGTATG--A-TAGATGTTGCGGGCCCCCAAGTCCAACTAGAATGGACAGGTTAGGCGCATGCGTTATGCTAGGCCAGATTGTGG-ACACCCACCCTTTCGGGTAG
Scytonema arcangeli M09	GG--CCTCGGCGCTCTGGGGTATR--A-TAGATGTTGCGGGCCCCCAAGTCCAACTAGAATGGACAGGTTAGGCGCATGCGTTATGCTAGGCCAGATTGTGG-ACACCCACCCTTTCGGGTAG
Scytonema hyalinum sp. 1 CMT-1BRIN-NPC30	?????????CTCAATGCAAATTTACGGTCGTTAACCATAAATGTTTTGTCTTGGACGAGCCATCTGTAGCGTCGATTCTGCGAGAATGCAATTTAGCCGTCATGGGTTTTTTTCGTCGAACGA
Scytonema hyalinum sp. 1 CMT-1BRIN-NPC31	?????????CTCAATGCAAATTTACGGTCGTTAACCATAAATGTTTTGTCTTGGACGAGCCATATGTAGCGTCGATTCTGCGAGAATGCAATTTAGCCGTCATGGGTTTTTTTCGCAAAACGA
Scytonema hyalinum sp. 1 CMT-1BRIN-NPC32	AAGTTTCGACTCAATGCAAATTTACGGTCGTTAACCATAAATGTTTTGTCTTGGACGAGCCATCTGTAGCGTCGATTCTGCGAGAATGCAATTTAGCCGTCATGGGTTTTTTTCGTCGAACGA
Scytonema hyalinum sp. 1 CMT-1BRIN-NPC39	AAGTTTCGACTCAATGCAAATTTACGGTCGTTAACTATAAATGTTTTGTCTTGGACGAGCCATATGTAGCGTCGATTCTGCGAGAATGCAATTTAGCCGTCATGGGTTTTTTTCGCAAAACGA
Scytonema hyalinum sp. 1 CMT-1SWIN-NPC17	?????????CTCAATGCAAATTTACGGTCGTTAACCATAAATGTTTTGTCTTGGACGAGCCATATGTAGCGTCGATTCTGCGAGAATGCAATTTAGCCGTCATGGGTTTTTTTCGTCGAACGA
Scytonema hyalinum sp. 1 EM3-HA15	?AGTTTCGACTCAATGCAAATTTACGGTCGTATACCATAAATGTTTTGTCTTGGACGAGCCATCTGTAGCGTCGATTCTGCGAGAATGCAATTTAGCCGTCATGGGTTTTTTTCGCAAAACGA
Scytonema hyalinum sp. 1 EM3-HA19	?AGTTTCGACTCAATGCAAATTTACGGTCGTATACCATAAATGTTTTGTCTTGGACGAGCCATCTGTAGCGTCGATTCTGCGAGAATGCAATTTAGCCGTCATGGGTTTTTTTCGCAAAACGA
Scytonema hyalinum sp. 1 EM3-HA20	?AGTTTCGACTCAATGCAAATTTACGGTCGTATACCATAAATGTTTTGTCTTGGACGAGCCATCTGTAGCGTCGATTCTGCGAGAATGCAATTTAGCCGTCATGGGTTTTTTTCGCAAAACGA
Scytonema hyalinum sp. 1 EM3-HA25	AAGTTTCGACTCAATGCAAATTTACGGTCGTATACCATAAATGTTTTGTCTTGGACGAGCCATCTGTAGCGTCGATTCTGCGAGAATGCAATTTAGCCGTCATGGGTTTTTTTCGCAAAACGA
Scytonema hyalinum sp. 1 EM3-Z1_c02	?AGTTTCGACTCAATGCAAATTTACGGTCGTTAACCATAAATGTTTTGTCTTGGACGAGCCATCTGTAGCGTGCATTCTGCGGGAATGCAATTTAGCCGTCATGGGTTTTTTTCGCAAAACGA
Scytonema hyalinum sp. 1 WJT4-NPBG1	AAGTTTCGACTCAATGCAAATTTACGGTCGTTAACCATAAATGTTTTGTCTTGGACGAGCCATCTGTAGCGTGCATTCTGCGAGAATGCAATTTAGCCGTCATGGGTTTTTTTCGTCGAACGA
Scytonema hyalinum sp. 1 FI5-JRJ03_	?AGTTTCGACTCAATGCAAATTTACGGTCGTTAACCATAAATGTTTTGTCTTGGACGAGCCATATGTAGCGTCGATTCTGCGAGAATGCAATTTAGCCGTCATGGGTTTTTTTCGCAAAACGA
Scytonema hyalinum sp. 1 WJT9-NPBG6A	AAGTTTCGACTCAATGCAAATTTACGGTCGTTAACTATAAATGTTTTGTCTTGGACGAGCCATATGTAGCGTCGATTCTGCGAGAATGCAATTTAGCCGTCATGGGTTTTTTTCGCAAAACGA
Scytonema hyalinum sp. 1 WJT9-NPBG6B	AAGTTTCGACTCAATGCAAATTTACGGTCGTTAACTATAAATGTTTTGTCTTGGACGAGCCATATGTAGCGTCGATTCTGCGAGAATGCAATTTAGCCGTCATGGGTTTTTTTCGCAAAACGA
Scytonema_hyalinum sp. 2 HK-05	AAGTTTCGACTCAATGCAAATTTACGGTCGTTAACCATAAATGTTTTGTCTTGGACGAGCCATCTGTAGCGTCGATTCTGCGAGAATGCAATTTAGCCGTCATGGGTTTTTTTCGCAAAACGA
Scytonema hyalinum sp. 3 HAF2-B2-C1	?AGTTTCGACTCAATGCAAATTTACGGTCGTTAACCATAAATGTTTTGTCTTGGACGAGCCATCTGTAGCGTCGATTCTGCGAGAATGCAATTTAGCCGTCATGGGTTTTTTTCGCAAAACGA
Scytonema hyalinum sp. 4 HTT-U-KK4	AAGTTTCGACTCAATGCAAATTTACGGTCGTTAACCATAAATGTTTTGTCTTGGACGAGCCATCTGTAGCGTCGATTCTGCGGGAATGCAATTTAGCCGTCATGGGTTTTTTTCGCAAAACGA
Scytonema hyalinum sp. 5 HA4185-MV1	?AGTTTCGACTCAATGCAGAATAAGGGTCGTTAACCATAAATGTTTTGTCTTGGACGAGCCATCTGTAGCATCGATTCTGCGAGAATGCAGTTTAGCCGTCATGGGTTTTTTTCGTCGAACGA
Scytonema hyalinum sp. 5 ATA-SAL-RM1	????TTCGACTCAATGCAGAATAAGGGTCGTTAACCATAAATGTTTTGTCTTGGACGAGCCATCTGTAGCATCGATTCTGCGAGAATGCAGTTTAGCCGTCATGGGTTTTTTTCGTCGAACGA
Scytonema_arcangeli Valle de Zapoltlan	?AGTTTCGACTCAATGCAAATTTATGGTCGTTAACCATAAATGTTTTGTCTTGGACGAGCCATCTGTAGCATCGATTCCGCGGGAATGCAGTTTAGCCGTCATGGG?????????????????
Scytonema arcangeli FL17-MK96	?AGTTTCGACTCAATGCAAATTTATGGTCGTTAACCATAAATGTTTTGTCTTGGACGAGCCATCTGTAGCATCGATTCCGCGGGAATGCAGTTTAGCCGTCATGGG?????????????????
Brasilonema_BZ_HDL_007	GAGTTTCGACTGCTCCGGGGTATG--A-TAGTAATCGCGGTCCCCTTTTCCAGAAGAATTGGACAGGATAGCGGCTCCGCGGGAGCGCGGCCAGGCTGAGG-ACACCCACCCTACTTGGTAG
Brasilonema_CR6-4B	?????????CTGCTCCGGGGTTGG--A-TAGTAATCGCGGTCCCCTTATCCAGAAGAATTGGACAGGATAGCGGCTTCGCGGAAGCGCGGCCAGGCCGAGG-ACACCCACCCTGCAAGGTAG
Brasilonema_CR6-5A	GAGTTTCGACTGCTCCGGGGAACG--A-TAGTAATCGCGGTCCCCTTTTCCAGAAGAATTGGACAGGATAGCGGCTTCGCGGAAGCGCGGCCAGGCCGAGG-ACACCCACCCTGCAAGGTAG
Brasilonema_KEN_MK50	GAGTTTCGACTGCTCCGGGGTATG--A-TAGTAATCGCGGTCCCCTTTTCCAGAAGAATTGGACAGGAAAGCGGCTTCGCGGAAGCGCGGCCAGGCCGAGG-ACACCCACCCTCCAAGGTAG
Brasilonema_M31_F20B	GAGTTTCGACTGCTCCGGGGTATG--A-TAGTAATCGCGGTCCCCTTTTCCAGAAGAGCTGGACAGGGCAGCGGCTCTGCGAGAACGCGGCCAGGCTGAGG-ACACCCACCCTCTCGGGTAG
Brasilonema_P09_MK13	GAGTTTCGACTGATCTGGGGTCGG--A-TGTTAGTCGCGGTCCCCTTTTCCAGAAGAATTGGACAGGGCAGCGGCTCTGCGAGAGCGCGGCCAGACTGAGG-ACACCCACCCTCTCGGGTAG
Brasilonema_PT5_MK70	GAGTTTCGACTGATCTGGGATCGG--A-TGTTAGTCGCGGTCCCCTTGTCCAGAAGAGCTGGACAGGGCAGCGGCTCTGCGAGAGCGCGGCCAGGCCGAGG-ACACCCACCCTGTCGGGTAG
Brasilonema_TH04_Ema	GAGTTTAGACTGATCTGGGGTAGG--A-TGTTAGTCGCGGTCCCCTTTTCCAGAAGAATTGGACAGGATAGCGGCTCTGCGAGAACGCGGCCAGGCCGAGG-ACACCCACCCTCTCGGGTAG
Scytonema hofmannii_PCC7110	GACCCTAGGTTGCTTTGGGGATGG--G-CGGTAGCTGCGGACCCCTTTTCCAGAAGAAATGGACAGGATTGGCGCCCGTGTGGGGCTAGGCCAGATTGTGG-ACACCAGTCTTATTCGGNAG
Scytonema hofmannii_FM5_MK45	????????????????????????????????????GCGGCCCCCTTTACCCAGAAGAAATGGACAGGATTGGCGCCCGTGTGGGGCTAGGCCAGATTGTGG-ACACCAGTCTTATCGGGCAG
Scytonema fritschii_UCFS22	G-TTCAGGACTGCTCTGGGGAAAA--G-CGGTAGTTGCGGACCCCCAATCCCAGATAGATTGGACAGGAAAGGCGTTTTTGCAGAGCTAGGCCAGGCCGTGG-ACACCCACCCTC????????
Scytonema fritschii_UCFS23	G-TTCTAGACTGCTCTGGGGTTGG--G-CGGTAGTTGCGGACCCCCAATCCCAGATAGATTGGACAGGAAAGGCGTTTCTGTGGAGCTAGGCCAGGCCGTGG-ACACC???????????????
