# Supplementary material for: Highly divergent 16S rRNA sequences in ribosomal operons of Scytonema hyalinum (Cyanobacteria)
Source: PLoS One. 2017 Oct 26;12(10):e0186393. doi: 10.1371/journal.pone.0186393 (PMC5658200; doi:10.1371/journal.pone.0186393)
Supplement: S4 Table — (PDF) [file pone.0186393.s004.pdf]

S4 Table. Summary of NCBI accession numbers for *rbc*LX, *rpo*C1, and *nif*D nucleotide sequences obtained in this study.

Strain	NCBI Accession		
	***rbc*LX**	***rpo*C1**	***nif*D**
Scytonema arcangeli M09	KY417041	KY423285	KY416993
Scytonema hyalinum sp. 5 ATA-SAL-RM1	KY417083	KY423327	KY417035
Scytonema hyalinum sp. 5 HA4185-MV1	KY417084	KY423328	KY417036
Scytonema arcangeli THH60	KY417042	KY423286	KY416994
Scytonema arcangeli Valle de Zapotitlan	KY417046	KY423290	KY416998
Scytonema hyalinum sp. 2 F10-MK28	KY417044	KY423288	KY416996
Scytonema hyalinum sp. 3 HAF2-B2-c1	KY417081	KY423325	KY417033
Scytonema hyalinum sp. 4 HTT-U-KK4	KY417082	KY423326	KY417034
Scytonema hyalinum sp. 1 PT10-MK84	KY417045	KY423289	KY416997
Scytonema hyalinum sp. 1 FI5-JRJ03	KY417048	KY423292	KY417000
Scytonema hyalinum sp. 1 EM3-Z1-c02	KY417049	KY423293	KY417001
Scytonema hyalinum sp. 1 EM3-HA19	KY417050	KY423294	KY417002
Scytonema hyalinum sp. 1 EM3-HA25	KY417051	KY423295	KY417003
Scytonema hyalinum sp. 1 EM3-HA15	KY417052	KY423296	KY417004
Scytonema hyalinum sp. 1 EM3-HA20	KY417053	KY423297	KY417005
Scytonema hyalinum sp. 1CMT-1SWIN-NPC17	KY417076	KY423320	KY417028
Scytonema hyalinum sp. 1 CXA108-5-BZ	KY417074	KY423318	KY417026
Scytonema hyalinum sp. 1 CNP2-Z1-c01	KY417077	KY423321	KY417029
Scytonema hyalinum sp. 1 CMT-1BRIN-NPC31	KY417075	KY423319	KY417027
Scytonema hyalinum sp. 1 WJT71-NPBG27	KY417085	KY423329	KY417037
Scytonema hyalinum sp. 1 WJT73-NPBGC	KY417086	KY423330	KY417038
Scytonema hyalinum sp. 1 WJT4-NPBG1	KY417078	KY423322	KY417030
Scytonema hyalinum sp. 1 WJT9-NPBG6A	KY417079	KY423323	KY417031
Scytonema hyalinum sp. 1 WJT9-NPBG6B	KY417080	KY423324	KY417032
Scytonema hyalinum sp. 1 DV1-KK3	KY417047	KY423291	KY416999
Brasilonema sp. CR06-4B/1	KY417056	KY423300	KY417008
Brasilonema sp. BZ-HDL-007	KY417055	KY423299	KY417007
Brasilonema sp. KEN-MK50	KY417059	KY423303	KY417011
Brasilonema sp. CR6-5A/1	KY417057	KY423301	KY417009
Brasilonema sp. M31-F20B	KY417058	KY423302	KY417010
Brasilonema sp. TH04-Ema	KY417061	KY423305	KY417013
Brasilonema sp. P09-MK13	KY417054	KY423298	KY417006
Brasilonema sp. PT5-MK70	KY417060	KY423304	KY417012
Scytonema stuposum M10-F15A	KY417066	KY423310	KY417018
Scytonema cf. *chiastum* M32-F26III	KY417064	KY423308	KY417016
Scytonema cf. *chiastum* F04-MK25	KY417062	KY423306	KY417014
Scytonema cf. *chiastum* GSE-NOS-MK14-07B	KY417063	KY423307	KY417015
Hassallia sp. CMT-1BRIN-NPC13	KY417069	KY423313	KY417021
Hassallia sp. CMT-1SZIN-NPC9	KY417070	KY423314	KY417022
Hassallia sp. CMT-2BRIN-HLNPC9	KY417071	KY423315	KY417023
Hassallia sp. CXA109-3-BZ	KY417073	KY423317	KY417025
Hassallia sp. CMT-3SWIN-NPC18	KY417072	KY423316	KY417024
Roholtiella mojaviensis WJT36-NPBG10 str. CCALA1051	KY417088	KY423331	KY417040
Roholtiella mojaviensis WJT36-NPBG5B str. CCALA 1052	KY417087	KY423332	KY417039
Scytonema sp. FM5-MK45	KY417065	KY423309	KY417017
Scytonematopsis sp. M07	KY417067	KY423311	KY417019
Scytonematopsis sp. PNG7-MK64	KY417068	KY423312	KY417020
